# Ability of Two Strains of Lactic Acid Bacteria To Inhibit Listeria monocytogenes by Spot Inoculation and in an Environmental Microbiome Context

**DOI:** 10.1128/spectrum.01018-22

**Published:** 2022-07-19

**Authors:** Priscilla Sinclair, M. Laura Rolon, Jingzhang Feng, Adrián F. Padín-López, Luke LaBorde, Jasna Kovac

**Affiliations:** a Department of Food Science, The Pennsylvania State University, University Park, Pennsylvania, USA; b Microbiome Center, The Pennsylvania State University, University Park, Pennsylvania, USA; University of Torino

**Keywords:** *Listeria monocytogenes*, biocontrol, lactic acid bacteria, inhibition, attached biomass, environmental microbiome, biofilms

## Abstract

We evaluated the ability of two strains of lactic acid bacteria (LAB) to inhibit L. monocytogenes using spot inoculation and environmental microbiome attached-biomass assays. LAB strains (PS01155 and PS01156) were tested for antilisterial activity toward 22 phylogenetically distinct L. monocytogenes strains isolated from three fruit packing environments (F1, F2, and F3). LAB strains were tested by spot inoculation onto L. monocytogenes lawns (10^8^ and 10^7^ CFU/mL) and incubated at 15, 20, 25, or 30°C for 3 days. The same LAB strains were also cocultured at 15°C for 3, 5, and 15 days in polypropylene conical tubes with L. monocytogenes and environmental microbiome suspensions collected from F1, F2, and F3. In the spot inoculation assay, PS01156 was significantly more inhibitory toward less concentrated L. monocytogenes lawns than more concentrated lawns at all the tested temperatures, while PS01155 was significantly more inhibitory toward less concentrated lawns only at 15 and 25°C. Furthermore, inhibition of L. monocytogenes by PS01156 was significantly greater at 15°C than higher temperatures, whereas the temperature did not have an effect on the inhibitory activity of PS01155. In the assay using attached environmental microbiome biomass, L. monocytogenes concentration was significantly reduced by PS01156, but not PS01155, when cocultured with microbiomes from F1 and F3 and incubated for 3 days at 15°C. Attached biomass microbiota composition was significantly affected by incubation time but not by LAB strain. This study demonstrates that LAB strains that may exhibit inhibitory properties toward L. monocytogenes in a spot inoculation assay may not maintain antilisterial activity within a complex microbiome.

**IMPORTANCE** Listeria monocytogenes has previously been associated with outbreaks of foodborne illness linked to consumption of fresh produce. In addition to conventional cleaning and sanitizing, lactic acid bacteria (LAB) have been studied for biocontrol of L. monocytogenes in food processing environments that are challenging to clean and sanitize. We evaluated whether two specific LAB strains, PS01155 and PS01156, can inhibit the growth of L. monocytogenes strains in a spot inoculation and in an attached-biomass assay, in which they were cocultured with environmental microbiomes collected from tree fruit packing facilities. LAB strains PS01155 and PS01156 inhibited L. monocytogenes in a spot inoculation assay, but the antilisterial activity was lower or not detected when they were grown with environmental microbiota. These results highlight the importance of conducting biocontrol challenge tests in the context of the complex environmental microbiomes present in food processing facilities to assess their potential for application in the food industry.

## INTRODUCTION

Listeria monocytogenes is one of the leading causes of foodborne illness-related deaths in the United States (1). Infections with L. monocytogenes result in listeriosis which causes an estimated 1,600 hospitalizations and 250 deaths each year (CDC, 2018) (1). L. monocytogenes is commonly found in cool- and wet-food processing facilities, where it can survive, grow (2), or form biofilms (3–6). Contamination with L. monocytogenes is particularly concerning in facilities that produce ready-to-eat food products that support growth of L. monocytogenes during storage and those that do not require cooking before consumption (7–12). Fresh whole produce, including tree fruit, is commonly consumed raw. While such produce generally does not support substantial growth of L. monocytogenes, the pathogen is capable of surviving under typical storage conditions, thus representing a food safety risk (13–15). Recent disease outbreaks and recalls of tree fruit due to L. monocytogenes contamination highlight the need for enhanced control of L. monocytogenes within the fruit packing environment (16, 17). The use of difficult-to-clean equipment in inaccessible areas renders chemical cleaning and sanitizing procedures less effective as a result of biofilm buildup over time (5, 18–20).

Multiple studies have shown that isolates of L. monocytogenes are capable of forming biofilms (3–6, 18, 21–23). Once the mature phase of biofilm development is reached, L. monocytogenes can be recurrently spread in the environment, increasing the risk for food contamination (3, 5, 18). In addition to L. monocytogenes, other members of the environmental microbiota found in tree fruit packing facilities, such as Pseudomonas (24), have been shown to form robust biofilms (25–30). Once L. monocytogenes is incorporated into a biofilm, an extracellular polymeric substance (EPS) matrix can provide a physical barrier that reduces sanitizer diffusion (31), which decreases the exposure of pathogens to lethal antimicrobial concentrations (19, 32–35).

In order to complement chemical cleaning and sanitizing of difficult-to-clean equipment and environments, biocontrol strains with antilisterial properties have previously been evaluated for application in food processing environments. Antilisterial activities have been attributed to (i) the production of secondary metabolites (e.g., bacteriocins), hydrogen peroxide, and organic acids, (ii) competitive exclusion due to competition for the same resources, or (iii) bacteriophage-mediated lysis (36–45). The potential for the use of biocontrol cultures of lactic acid bacteria (LAB) to reduce pathogen levels has been widely explored for use in food preservation and safety applications (38, 42, 45–49). LAB have been also shown to significantly inhibit L. monocytogenes *in vitro* (35, 38, 49–51). For example, Zhao et al. (52) isolated and evaluated two LAB strains for their effectiveness in inhibiting L. monocytogenes in poultry processing facility drains (51–53). The two LAB strains (152 and C-1-152) were reported to successfully inhibit the growth up to 4.1 log CFU/mL of *Listeria* spp. (53). However, it is not clear whether these strains would perform as well in the presence of environmental microbiomes found in other food processing facilities, such as tree fruit packing facilities.

Here, we evaluated whether the two LAB strains isolated by Zhao et al. (52), PS01155 (i.e., C-1-152; ATCC PTA-4761) and PS01156 (i.e., 152; ATCC PTA-4759), (i) exhibit antilisterial activity toward L. monocytogenes isolates previously collected from tree fruit packing facilities and (ii) maintain antilisterial activity against a persistent strain of L. monocytogenes when cocultured with microbiomes obtained from the same tree fruit packing facilities using an *in vitro* model system.

## RESULTS

### Strain PS01155 was identified as Enterococcus faecium and strain PS01156 as Enterococcus lactis based on whole-genome sequence analysis.

Strains PS01155 and PS01156, previously identified by 16S rRNA sequencing by Zhao et al. (52) as Lactococcus lactis subsp. *lactis* C-1-152 and Enterococcus durans 152, respectively, were purchased from the American Type Culture Collection (ATCC). These strains were selected for the levels study due to previous reports of their ability to significantly reduce L. monocytogenes over the course of 34 weeks in the drains of a poultry processing facility (53). Whole-genome sequencing (WGS) was used to confidently determine the taxonomic identity of the strains purchased from ATCC. Sequencing reads of PS01155 and PS01156 genomes assembled in 103 and 159 contigs, respectively. The average assembly coverages were 400 and 442, and the total lengths of the draft assemblies were 2,767,103 and 2,852,918 bp, respectively.

**FIG 1:**
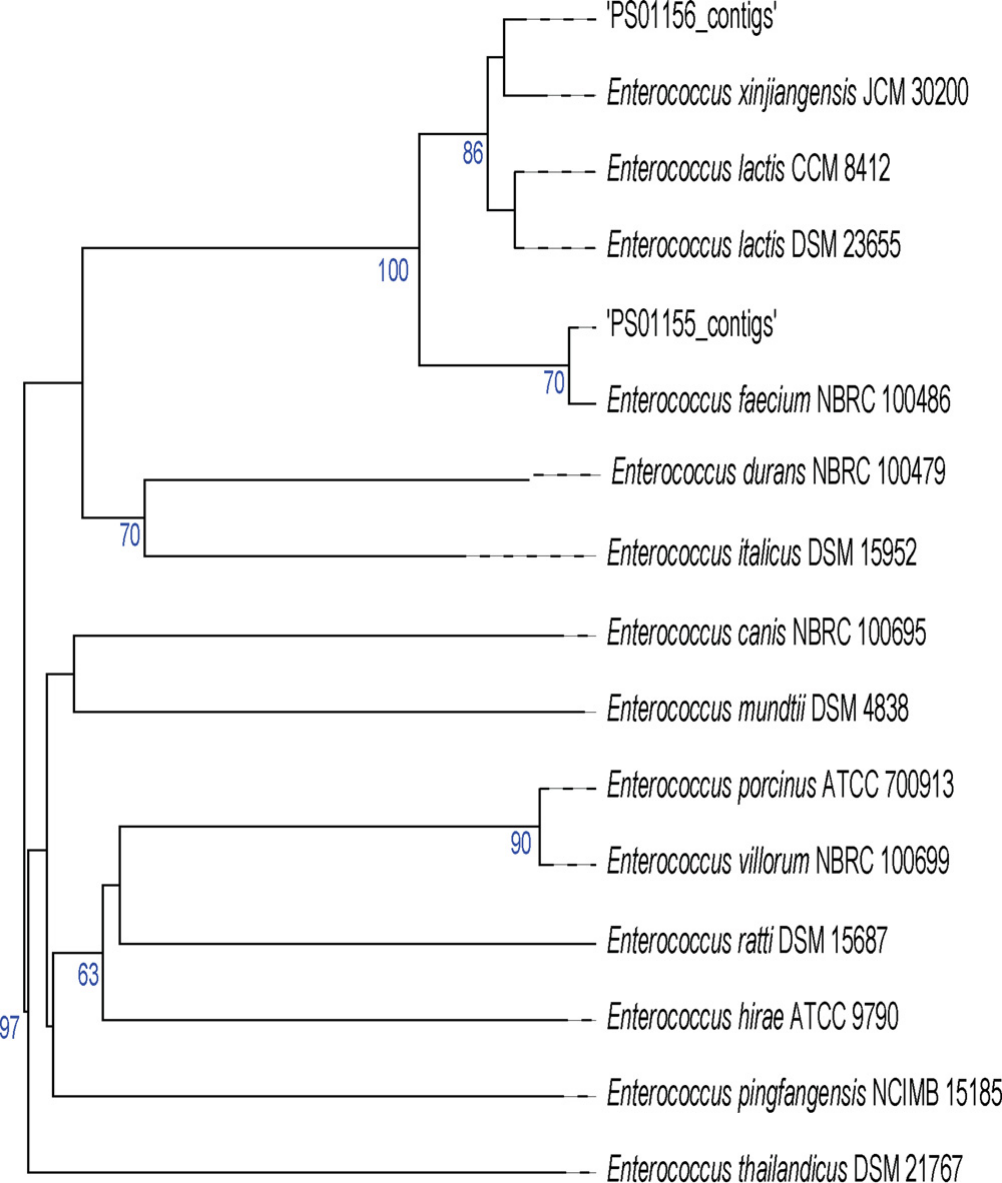
Phylogenetic tree based on and whole-genome sequence for lactic acid bacteria PS01155 and PS01156 as produced by Type (Strain) Genome Server. The branch lengths are scaled in terms of the Genome BLAST Distance Phylogeny (GBDP) distance formula *d*_5_. The numbers above branches are GBDP pseudo-bootstrap support values of >60% from 100 replications, with an average branch support of 43.1%. The tree was rooted at the midpoint.

The taxonomic identities of PS01155 and PS01156 were determined using the Type (Strain) Genome Server (54). Strain PS01155 was identified as Enterococcus faecium (not Lactococcus lactis), and strain PS01156 was identified as Enterococcus lactis (not Enterococcus durans) (Fig. 1). The identification of PS01155 and PS01156 had distance (*d*_4_) scores of 98.0 and 87.5, respectively, which are above the cutoff of 70 that is required for a confident taxonomic identification using a draft genome (54). PS01156 was also shown to be closely related to Enterococcus xinjiangensis (Fig. 1); however, a recent study showed a high degree of similarity between the type strains of Enterococcus lactis and *Enterococcus xinjiangensis*, suggesting that they represent the same species (55).

The draft genomes of strains PS01155 and PS01156 were submitted to BAGEL4 web server to detect bacteriocin encoding genes (56). In the assembly of the strain PS01155, BAGEL4 detected genes encoding enterocin B (accession no. WP_002295295.1) enterolysin A (accession no. WP_005877003.1), enterocin P (accession no. WP_010733280.1), and enterocin A (accession no. WP_002304799.1) (see Table S1 in the supplemental material). In the assembly of the strain PS01156, BAGEL4 detected genes encoding enterocin P (accession no. WP_002291094.1), enterocin L50b (accession no. WP_002293183.1), enterocin L50a (accession no. WP_236918740.1), enterolysin A (accession no. WP_002293508.1), and UviB (accession no. WP_002342148.1) (Table S1).

### Preliminary safety assessment of tested LAB strains.

Given that strains considered for application as biological controls should be safe for humans, we carried out preliminary assessment of safety for strains PS01155 and PS01156. Specifically, we assessed their hemolytic activity, MICs of selected antimicrobials, and the presence of virulence genes. Hemolysis tests showed no hemolytic activity of the two tested strains. Sensititre Gram Positive GPN3F plates were used to determine the MICs of 18 antimicrobials, and results were interpreted as resistant (R), intermediate (I), or susceptible (S) by following the CLSI M100-ED32 (2022) guideline for *Enterococcus* (Table 1) (57). Both strains were susceptible to first-line drugs for treatment of enterococcal infections, ampicillin, penicillin, and vancomycin (Table 1). MICs of these three antibiotics, as well as other antibiotics of lesser or no clinical relevance, are reported in Table 1. To assess the presence of putative virulence genes, the assembled genomes of PS01155 and PS01156 were submitted to the Center for Genomics Epidemiology web server for analysis with VirulenceFinder using default settings for *Enterococcus* species (58). Putative virulence genes, including collagen adhesin-encoding *acm* (accession no. CP003351.1) (59) and cell wall adhesin-encoding *efaA*fm (accession no. AF042288.1) (60), were detected in the assembled genomes of strains PS01155 and PS01156.

**TABLE 1:** MICs of selected antimicrobials for LAB strains PS01155 and PS01156

Antimicrobial	MIC (mg/L) (S/I/R)^*a*^	
	PS01155	PS01156
Erythromycin	4 (I)	>4 (R)
Clindamycin^*b*^	0.5	>2
Quinupristin/dalfopristin	1 (S)	2 (I)
Daptomycin	8 (R)	>8 (R)
Vancomycin	<1 (S)	<1 (S)
Tetracycline	<2 (S)	<2 (S)
Ampicillin	0.5 (S)	2 (S)
Gentamicin^*b*^	8	8
Levofloxacin	4 (R)	0.5 (R)
Linezolid	2 (S)	4 (I)
Ceftriaxone^*b*^	>64	>64
Streptomycin^*b*^	1,000	1,000
Penicillin	4 (S)	8 (S)
Rifampin	>4 (R)	>4 (R)
Gatifloxacin	<1 (S)	<1 (S)
Ciprofloxacin	2 (I)	2 (I)
Trimethoprim-sulfamethoxazole^*b*^	1/19	<0.5/9.5
Oxacillin + 2% NaCl^*b*^	>8	>8

a S, susceptible; I, intermediate; R, resistant. Resistance was interpreted using the CLSI M100-ED32 (2022) guideline for *Enterococcus.* When the culture grew in wells with all tested antibiotic concentrations present in the Sensititre plate, the MIC is reported as greater than the highest tested concentration.

b An MIC breakpoint is not defined and/or this antibiotic is not clinically effective, as per the CLSI M100-ED32 (2022) guideline for *Enterococcus*.

### Strains PS01155 and PS01156 exhibited robust inhibition of phylogenetically diverse L. monocytogenes strains grown at different concentrations and incubation temperatures.

**FIG 2:**
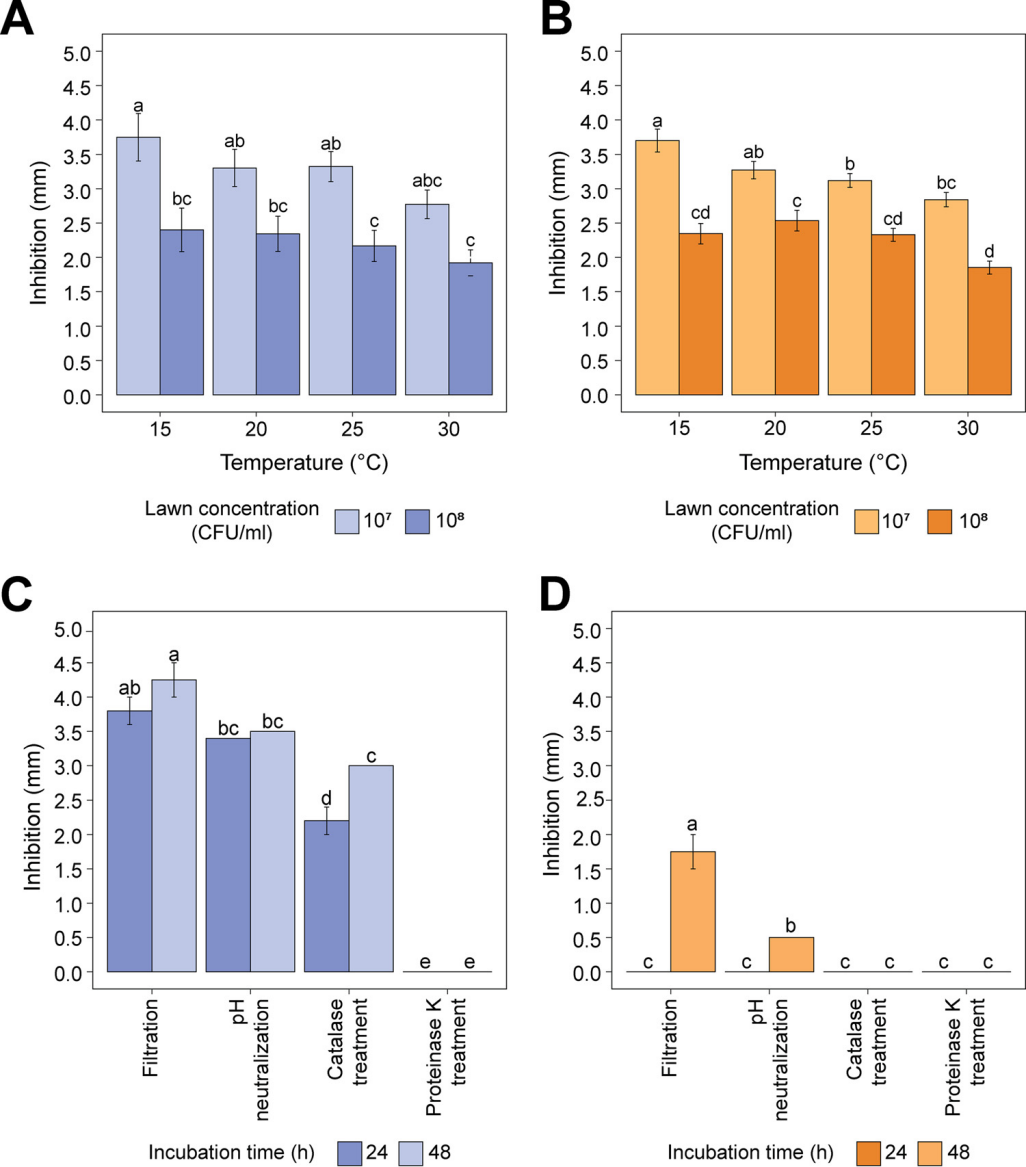
Inhibition of L. monocytogenes strains by (A) PS01155 and (B) PS01156 at 20, 25, and 30°C (*n* = 22) and at 15°C (*n* = 21) (*n* represents the number of L. monocytogenes strains tested at each temperature) using the spot inoculation assay on BHI agar plates. Light bars represent the average zone of inhibition observed on 10^7^CFU/mL L. monocytogenes lawns, and dark bars represent the average zone of inhibition observed on the 10^8^CFU/mL L. monocytogenes lawns at 15, 20, 25, and 30°C, with standard error bars. Average zone of inhibition by supernatants of PS01155 (C) and PS01156 (D) after filtration, pH neutralization, catalase, and proteinase K treatment to determine the nature of the inhibition. Dark bars represent the supernatant obtained from 24-h cultures grown in MRS broth, and light bars represent the supernatant obtained from 48-h cultures grown in MRS broth. For each panel, letters represent significant differences between treatments (*P* < 0.05) as determined by one-way ANOVA followed by Tukey’s HSD *post hoc* test.

Strains PS01155 and PS01156 inhibited the growth of 22 L. monocytogenes isolates collected from tree fruit packing facilities (Table 2), as determined using a spot inoculation assay (Fig. 2A and B). PS01155 had a significantly stronger inhibition of less concentrated L. monocytogenes lawns (10^7^ CFU/mL versus 10^8^ CFU/mL) when incubated at 15°C or 25°C (*P* = 6.72 × 10^−7^) (Fig. 2A). PS01156 had a significantly stronger inhibition of the 10^7^-CFU/mL than the 10^8^-CFU/mL L. monocytogenes lawns when incubated at any of the tested temperatures (*P* < 2 × 10^−16^) (Fig. 2B). Strain PS01156 produced a significantly greater inhibition of L. monocytogenes at 15°C than at 25 and 30°C for the lawns inoculated with 10^7^ -CFU/mL L. monocytogenes (Fig. 2B). Temperature did not have a significant effect on the ability of the strain PS01155 to inhibit L. monocytogenes lawns at either concentration (Fig. 2A).

**TABLE 2:** Bacterial strains used in this study

Species	Strain	CC/ST^*a*^	Isolation source and date (mo/day/yr)	Reference
L. monocytogenes	CFSAN64020/PS01273	ST1515	Tree fruit packing facility F1, 3/26/17	94
	CFSAN62940/PS01274	ST1513	Tree fruit packing facility F3, 1/23/17	94
	CFSAN62927/PS01275	CC5	Tree fruit packing facility F3, 1/23/17	94
	CFSAN68750/PS01276	CC433/ST1516	Tree fruit packing facility F3, 3/7/17	94
	CFSAN62813/PS01277	CC37	Tree fruit packing facility F2, 3/7/17	94
	CFSAN62927/PS01278	CC5	Tree fruit packing facility F3, 1/13/17	94
	CFSAN58403/PS01279	CC379	Tree fruit packing facility F1, 11/17/16	94
	CFSAN62934/PS01280	ST1514	Tree fruit packing facility F3, 1/23/17	94
	CFSAN58407/PS01281	ST1509	Tree fruit packing facility F1, 11/17/16	94
	CFSAN62899/PS01282	ST1511	Tree fruit packing facility F1, 2/6/17	94
	CFSAN58390/PS01283	ST392	Tree fruit packing facility F1, 3/16/17	94
	CFSAN62947/PS01284	CC369/ST374	Tree fruit packing facility F3, 1/23/17	94
	CFSAN58387/PS01285	CC331	Tree fruit packing facility F1, 11/7/16	94
	CFSAN62900/PS01286	ST1512	Tree fruit packing facility F1, 2/6/17	94
	CFSAN58391/PS01287	CC288/ST323	Tree fruit packing facility F1, 11/7/16	94
	CFSAN56234/PS01288	ST1052	Tree fruit packing facility F1, 10/19/16	94
	CFSAN65257/PS01289	CC217/ST217	Tree fruit packing facility F1, 3/22/17	94
	CFSAN58399/PS01291	CC4/ST219	Tree fruit packing facility F1, 11/7/16	94
	CFSAN68749/PS01292	ST489	Tree fruit packing facility F3, 7/31/17	94
	CFSAN62942/PS01293	ST1510	Tree fruit packing facility F3, 1/28/17	94
	CFSAN56268/PS01294	CC1320/ST1507	Tree fruit packing facility F2, 3/22/17	94
	CFSAN56291/PS01295	ST1003	Tree fruit packing facility F2, 10/19/16	94
Lactococcus lactis subsp. *lactis*	C-1-92/PS01155		Floor drain of a food processing plant	52
Enterococcus durans	152/PS01156		Floor drain of a food processing plant	52

a Clonal complex (CC) and sequence type (ST) were obtained from reference 94.

### Characterization of antilisterial activity of PS01155 and PS01156.

To determine the nature of the inhibition, strains PS01155 and PS01156 were grown at 35°C in Brain Heart Infusion (BHI) broth, and the supernatants were tested for inhibition of L. monocytogenes. To assess the inhibitory effect of organic acids, hydrogen peroxide, and proteinaceous compounds produced by the two LAB strain, the cell-free supernatants were neutralized, treated with catalase, and proteinase K, and applied to lawns of L. monocytogenes after each treatment step. Filtered supernatants of strains PS01155 and PS01156 grown in BHI broth did not inhibit L. monocytogenes when tested on 10^7^-CFU/mL lawns of L. monocytogenes strain PS01273 in two independent experiments. Hence, additional growth temperatures and media that have been reported to induce the production of bacteriocins (61) were used to grow the two LAB strains (Table S2). Additionally, conditions reported in references 62 and 63 were used to assess bacteriocin production by strain PS01156 (Table S2).

All treated supernatants from strain PS01155 incubated for 24 and 48 h in De Mann, Rogosa, and Sharpe (MRS) broth (pH 6.2) inhibited the lawn of L. monocytogenes strain PS01273, except in the case of the proteinase K treatment. This confirmed that the inhibition of L. monocytogenes by the neutralized, hydrogen peroxide-free supernatant was due to proteinaceous compounds (Fig. 2C). Further, the zone of inhibition significantly decreased after the removal of organic acids and hydrogen peroxides (*P* = 6.54 × 10^−8^) (Fig. 2C). This suggests that inhibition due to proteinaceous compounds was not the only mechanism by which strain PS01155 inhibited L. monocytogenes. The supernatant of PS01156 inhibited L. monocytogenes lawns only when the strain was grown in MRS (pH 6.2) at 37°C for 48 h (*P* = 6.05 × 10^−6^). However, the inhibition was lost after treatment with catalase (Fig. 2D), suggesting that the inhibition observed was not due to proteinaceous compounds, such as bacteriocins. PS01156 was grown under additional growth conditions to induce bacteriocin production (Table S2). PS01156 was grown in two independent experiments and did not inhibit the L. monocytogenes lawn; thus, the antilisterial activity cannot be attributed to proteinaceous compounds under the tested conditions.

### The antilisterial activity of PS01155 and PS01156 was diminished in an attached biomass grown from an environmental microbiome collected from tree fruit packing facilities.

We further tested whether the presence of environmental microbiome present in tree fruit packing facilities could affect the antilisterial properties of PS01155 and PS01156, using a model attached-biomass system. PS01155 or PS01156 (~10^7^ CFU/mL) and L. monocytogenes strain PS01273 (~10^5^ CFU/mL) were inoculated with composite environmental microbiome suspensions collected from three tree fruit packing facilities (F1, F2, and F3). A positive-control sample included the environmental microbiome suspension and L. monocytogenes, and a negative-control sample included only the environmental microbiome. In the composite microbiome suspensions before addition of L. monocytogenes or lactic acid bacteria, the aerobic plate counts (mean ± standard deviation) were 6.32 ± 0.48, 5.40 ± 0.26, and 5.69 ± 0.44 log_10_ CFU/mL for F1, F2, and F3, respectively. L. monocytogenes concentrations, quantified using the Most Probable Number (MPN) method, were <1.52, 3.68 ± 0.37, and 1.55 ± 0.66 log_10_ MPN/mL for F1, F2, and F3, respectively.

**FIG 3:**
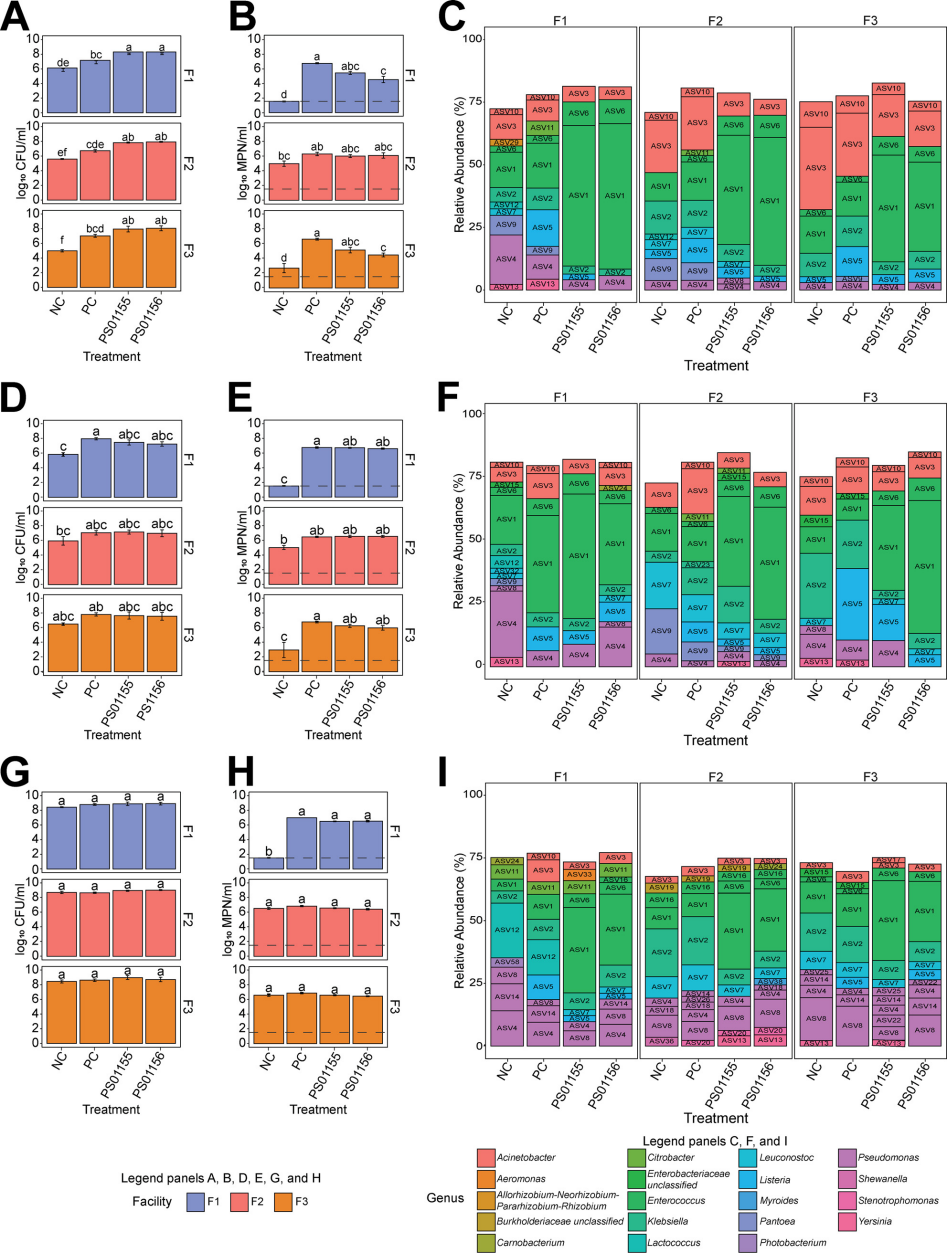
Aerobic plate counts in the attached biomass grown for 3 (A), 5 (D), and 15 (G) days and L. monocytogenes concentration in the attached biomass grown for 3 (B), 5 (E), and 15 (H) days from environmental microbiomes collected from facilities F1, F2, and F3. NC, negative control; PC, positive control. Bars are color coded by facility, and the error bars represent standard errors. For each panel, letters represent significant differences between treatments (*P* < 0.05). Microbiota composition of the attached biomass grown for 3 (C), 5 (F), and 15 (I) days. Bars represent the relative abundance of the ASVs with a relative abundance above 2% and are color coded by the assigned taxonomic genus.

Assays were incubated for 3 and 5 days without reapplication of PS01155 or PS01156. L. monocytogenes and aerobic mesophilic microorganisms present in the attached biomass were quantified after incubation. Statistical significance was assessed using one-way analysis of variance (ANOVA) for each time point, followed by Tukey’s honestly significant difference (HSD) test. After 3 days of incubation, the attached biomass had significantly higher concentration of aerobic mesophilic organisms for treatments that included the addition of strains PS01155 and PS01156 compared to the negative control, regardless of the facility from which a microbiome sample originated (*P* = 1.0 × 10^−10^) (Fig. 3A). However, the L. monocytogenes concentration in the attached biomass was significantly reduced when cocultured with strain PS01156 and the microbiotas collected from F1 and F3, compared to the positive control (*P* = 1.85 × 10^−10^) (Fig. 3B). In samples that included the microbiome from F1, the addition of strain PS01155 or PS01156 reduced L. monocytogenes by 1.29 and 2.19 log_10_ CFU/mL, respectively (Fig. 3B). In samples that included the microbiome from F2, the addition of PS01155 or PS01156 reduced L. monocytogenes by 0.29 or 0.211 log_10_ CFU/mL, respectively, but the reduction was not statistically significant compared to the positive control (Fig. 3B). In samples that included the microbiome from F3, the addition of PS01155 or PS01156 reduced L. monocytogenes by 1.48 or 2.14 log CFU/mL, respectively (Fig. 3B).

The microbiota composition of samples incubated for 3 days and treated with PS01155 or PS01156 showed a predominance of amplicon sequence variant 1 (ASV1) (*Enterococcus*) compared to the positive-control or negative-control treatments, regardless of the source of the microbiota (Fig. 3C). The sequence of ASV1 was identical to the 16S rRNA sequence obtained from the assembled genomes of both PS01155 and PS01156, suggesting that it may be a marker of these LAB strains. The relative abundance of ASV1 was lower in the samples that were cocultured with the microbiomes collected from F2 or F3 than in those with the microbiome from F1, suggesting that the added LAB strains most effectively attached to the test surface in assays with the microbiome collected from F3.

After 5 days of incubation, there was no significant difference between the concentrations of aerobic mesophilic organisms in the negative-control samples and those treated with PS01155 or PS01156, with the exception of samples that included the microbiota from F1 (*P* = 0.116) (Fig. 3D). Further, the concentration of L. monocytogenes was not significantly different in samples to which PS01155 or PS01156 was added compared to the positive control, regardless of the origin of the microbiome samples (Fig. 3E). The microbiota composition of samples incubated for 5 days and treated with PS01155 or PS01156 showed a high predominance of ASV1 (*Enterococcus*) compared to the positive-control and negative-control treatments when cocultured with the microbiota of F2 or F3 (Fig. 3F). However, the relative abundance of ASV1 was lower than after 3 days of incubation for the same treatment, with the exception of PS01156 when cocultured with the environmental microbiota of F1. Interestingly, the positive control of F1 incubated for 5 days showed a high relative abundance of ASV1 as well, suggesting that *Enterococcus* was present in the microbiome of F1. Other ASVs increased in relative abundance after 5 days of incubation compared to 3 days of incubation. Specifically, the relative abundance of ASV2 (Klebsiella) and ASV4 (Pseudomonas) increased in samples that were incubated for 5 days compared to samples incubated for 3 days (Fig. 3F).

To better assess the effect of PS01155 and PS01156 in a long-term application, we carried out a 15-day attached-biomass assay with repeated application of PS01155 and PS01156 every 5 days. We found no significant differences in the aerobic plate counts on day 15 for any treatment and facility microbiome (*P* = 0.470) (Fig. 3G). Further, the concentration of L. monocytogenes was not significantly different in samples to which PS01155 or PS01156 was added, regardless of the origin of the microbiome samples, compared to the positive control (Fig. 3H). The microbiota composition of samples incubated for 15 days and treated with PS01155 or PS01156 showed a predominance of ASV1 (*Enterococcus*), compared to the positive- and negative-control treatments, regardless of the source of the microbiota (Fig. 3I). However, the relative abundance of ASV1 was lower than after 3 and 5 days of incubation for the same treatments, with the exception of the addition of PS01156 to the microbiota of F3. Other ASVs increased in relative abundance in the 15-day experiment compared to the 3- and 5-day experiment. Specifically, the relative abundance of ASVs from the genus Pseudomonas (ASV4, ASV8, ASV14, and ASV25) increased in samples that were incubated for 15 days compared to those incubated for 3 and 5 days (Fig. 3I).

### Attached microbiota composition significantly differed among 3-, 5-, and 15-day assay endpoints.

**FIG 4:**
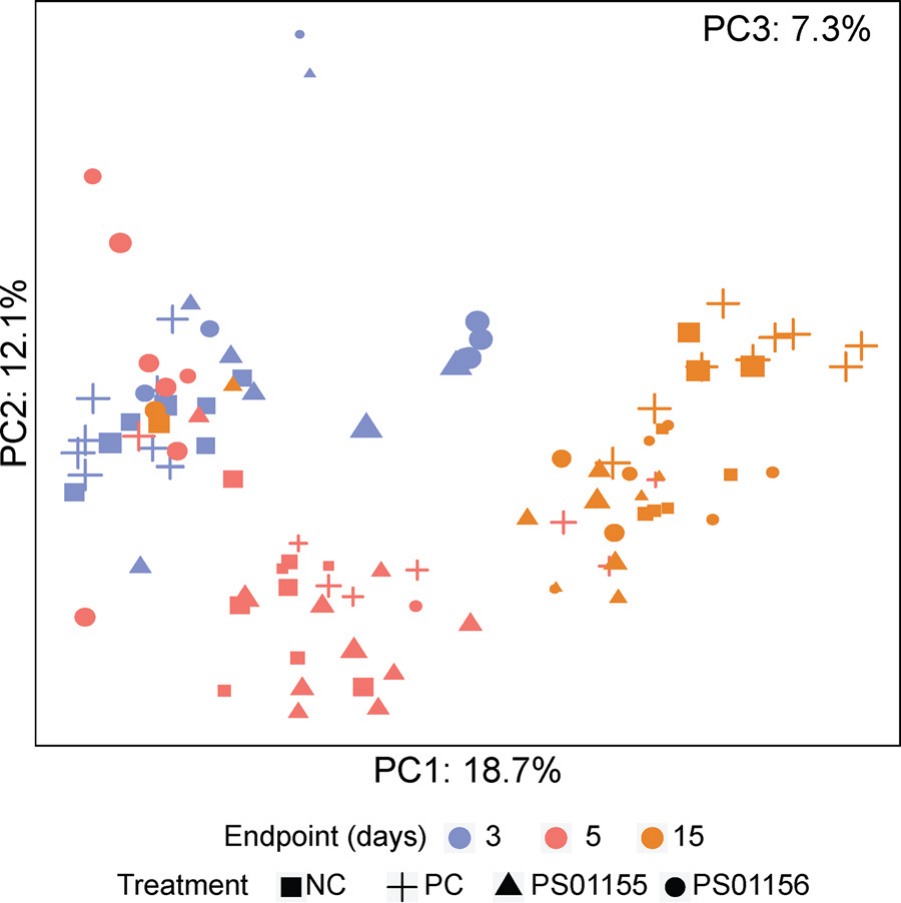
Principal-component analysis plot for the microbiota composition of the attached biomass grown for 3, 5, and 15 days. Each point represents the bacterial composition of one sample. The samples are color coded by growth period, where purple represents the attached-biomass composition for 3-day assay, pink represents a 5-day assay, and orange represents a 15-day assay with repeated culture addition. Squares indicate negative-control (NC) samples, plus symbols indicate positive-control (PC) samples, triangles indicate samples with added PS01155, and circles indicate samples with added PS01156. The size of the symbols represents the third principal component.

Principal-component analysis (PCA) was used to evaluate the similarity in the overall microbiota composition of the attached biomass samples by incubation time and facility. The first two principal components (PCs) explained 30.8% of the variance in the data (Fig. 4). There was a clear clustering of samples by incubation time (i.e., 3-, 5-, and 15-day experiments) (Fig. 4), but no observed clustering by facility or the addition of PS01155 or PS01156 (data not shown). Permutational multivariate analysis of variance (PERMANOVA) determined that the microbiota composition of the samples was not significantly different, regardless of the microbiome origin (F1, F2, or F3) (Table 3). However, the microbiota composition of samples was significantly different when incubation times were compared (3, 5, or 15 days), when positive-control treatment was compared against PS01155 treatment, and when PS01155 and PS01156 treatments were compared (Table 3).

**TABLE 3:** Differences in microbiota composition in attached biomass samples, as determined by PERMANOVA

Condition	Comparison^*a*^	Degrees of freedom	Sum of squares	F model	*R* ^2^	*P* ^*b*^
Growth time	3D vs. 5D	1	6,495	5.3345	0.07922	**0.001**
	5D vs. 15D	1	11,900	11.637	0.14431	**0.001**
	3D vs. 15D	1	13,995	13.18	0.17301	**0.001**

Facility	F1 vs. F3	1	671	0.5118	0.00781	0.989
	F1 vs. F2	1	1,187	0.92004	0.01417	0.511
	F3 vs. F2	1	1,238	0.95412	0.01447	0.453

Treatment	NC vs. PC	1	1,388	1.2873	0.02612	0.165
	NC vs. PS01155	1	1,353	1.125	0.02244	0.283
	NC vs. PS01156	1	2,085	1.5453	0.03183	0.066
	PC vs. PS01155	1	2,561	2.125	0.04156	**0.012**
	PC vs. PS01156	1	1,566	1.1584	0.02406	0.237
	PS01155 vs. PS01156	1	2,469	1.6764	0.03375	**0.029**

a 3D, 5D, and 15D indicate 3-day, 5-day, and 15-day attached-biomass assays. NC, negative control; PC, positive control.

b *P* value after Bonferroni correction. Boldface indicates statistically significant differences (*P* < 0.05).

**FIG 5:**
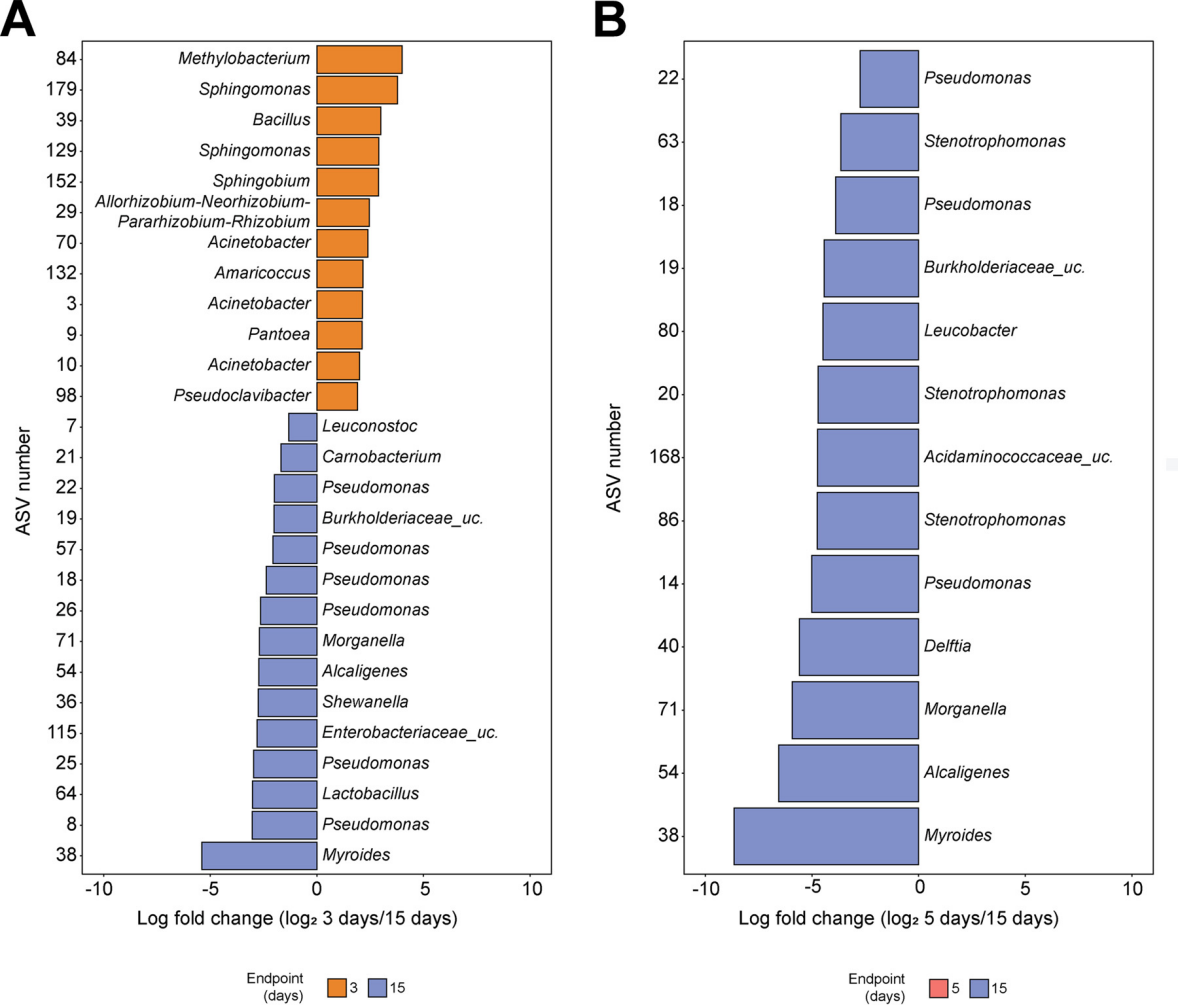
Differentially abundant taxa identified in 3-, 5-, and 15-day attached biomass. All samples (i.e., those treated with PS01155 and PS01156 and the positive and negative controls) were merged by day of experiment. The *x* axis represents the log fold change in relative abundance for ASVs that were differentially abundant (*P* < 0.05) and had an effect size above 1, calculated using ALDEx2. The color of the bars represents the experimental endpoint with increased relative abundance of each ASV. Each bar has a label corresponding to the assigned taxonomic genus for each ASV.

Differential abundance analysis was performed using ALDEx2 to identify ASVs that may be significantly differentially abundant among endpoint samples from 3-, 5-, and 15-day assays. No differentially abundant taxa were identified when the microbiota composition of samples incubated for 3 days was compared to that of samples incubated for 5 days. However, when the microbiota composition of samples incubated for 3 days was compared to that of samples incubated for 15 days, ALDEx2 identified 12 taxa (ASV84, *Methylobacterium*; ASV179 and ASV129, *Sphingomonas*; ASV39, *Bacillus*; ASV152, *Sphingobium*; ASV29, *Rhizobium*; ASV70, ASV3, and ASV10, Acinetobacter; ASV132, *Amaricoccus*; ASV9, *Pantoea*; and ASV98, *Pseudoclavibacter*) with a significantly higher relative abundance in samples incubated for 3 days and 15 taxa (ASV7, *Leuconostoc*; ASV21, *Carnobacterium*; ASV22, ASV57, ASV18, ASV26, ASV25, and ASV8, Pseudomonas; ASV19, *Burkholderiaceae* unclassified; ASV71, *Morganella*; ASV54, *Alcaligenes*; ASV36, *Shewanella*; ASV115, *Enterobacteriaceae* unclassified; ASV64, *Lactobacillus*; and ASV38, *Myroides*) with a significantly higher relative abundance in samples incubated for 15 days (Fig. 5A). Further, when samples incubated for 5 days were compared with samples incubated for 15 days, 13 taxa had a significantly higher relative abundance in samples incubated for 15 days (ASV22, ASV18, and ASV14, Pseudomonas; ASV63, ASV20, and ASV86, *Stenotrophomonas*; ASV19, unclassified *Burkholderiaceae*; ASV80, *Leucobacter*; ASV168, unclassified *Acidaminococcaceae*; ASV40, *Delftia*; ASV71, *Morganella*; ASV54, *Alcaligenes*; and ASV38, *Myroides*) (Fig. 5B).

## DISCUSSION

### Putative bacteriocins produced by strains PS01155 and PS01156.

Strain PS01155 was identified as Enterococcus faecium and strain PS01156 was identified as Enterococcus lactis based on WGS data analysis, which differed from the taxonomy previously reported by ATCC and Zhao et al. (52). Given that Zhao et al. (52) identified their isolates using 16S rRNA sequencing, it is likely that the identification was not conclusive. Further, in the 12 years since the isolates were first identified, an increased number of assembled genomes have been submitted to NCBI, which could have allowed us to obtain a more reliable identification. Further, it remains possible that there was a contamination issue at ATCC or that an incorrect isolate was sent to ATCC.

The web server BAGEL4 and BLAST analysis detected genes encoding enterocin B, enterolysin A, enterocin P, and enterocin A in the genome of E. faecium strain PS01155. *Enterococcus* spp. typically produce more than one bacteriocin (64), which is consistent with the detection of genes associated with the production of multiple bacteriocins in the genome of strain PS01155. Enterocin B and enterocin A are class IIa bacteriocins, which is the most common class of bacteriocins produced by *Enterococcus* spp. These bacteriocins are also known for their antilisterial activity (64). E. faecium is the species that is most commonly reported as a producer of class IIa bacteriocins (65, 66). Within class IIa, enterocin A has been identified as one of the most potent bacteriocins (67–69). Strains that produce enterocin A typically also produce enterocin B and other bacteriocins, which is in agreement with our genomic analyses results (70, 71). Enterolysin A is a class III bacteriocin which inactivates cells by degrading their cell wall structure, leading to lysis, but has not been shown to consistently inhibit *Listeria* spp. (66). The detection of bacteriocin-associated genes was consistent with the phenotypic results obtained using a spot inoculation assay in which the strain PS01155 inhibited all tested L. monocytogenes strains. To evaluate whether the inhibition was due to bacteriocin production by PS01155, the putative bacteriocins (i.e., inhibitory proteinaceous compounds) were partially isolated from supernatant of PS01155 after growth in MRS for both 24 and 48 h. The observed inactivation of the antimicrobial activity by proteinase K suggested that the inhibition previously observed was due to substances that were proteinaceous, such as bacteriocins (61).

Analysis of the genome of PS01156 resulted in identification of genes associated with the bacteriocins enterocin P, enterocin L50b, enterocin L50a, enterolysin A, and UviB. Enterocin P and UviB are class IIa bacteriocin; while enterocin L50b and enterocin L50a are class II leaderless bacteriocins (72–74). Enterocin P, UviB, and enterocin L50b have been reported to have antimicrobial activity against L. monocytogenes (66). Consistent with the detection of bacteriocin genes, strain PS01156 inhibited all 22 tested L. monocytogenes strains in the spot inoculation assay. However, at all tested growth temperatures, incubation times, and media (other than growth in MRS at 6.2 pH for 48 h), the filtered supernatant did not inhibit L. monocytogenes strain PS01273. The supernatant of PS01156 grown in MRS at 6.2 pH for 48 h exhibited only weak inhibition. Furthermore, once the hydrogen peroxide was removed, there was no inhibition observed. These results of the partial isolation of bacteriocins from PS01156 supernatant were inconsistent with reports of successful isolation of bacteriocins from the strain PS01156, previously reported as *E. durans* 152 (62). The lack of antilisterial activity also conflicted with the reports of Cintas et al. (63) and remains unexplained. Further research is needed to identify the optimal conditions for the production of bacteriocins by PS01156.

### Preliminary safety assessment of PS01155 and PS01156.

The genus *Enterococcus* is known to contain opportunistic human pathogen strain (75). The presence of putative virulence factors genes may reduce the potential for the application of PS01155 and PS01156 as agents of biological control in food processing facilities. We detected virulence factor genes *acm* (adhesion to collagen) and *efa*Afm (adhesion-like endocarditis antigens) in the assembled genomes of PS01155 and PS01156. Further studies are needed to determine whether the virulence genes are biologically functional and expressed. PS01155 and PS01156 were both resistant to the highest concentrations of ceftriaxone, rifampin, and oxacillin present in the Sensititre plates; however, these antibiotics are not of clinical relevance for treatment of *Enterococcus* infections. Importantly, the tested strains did not exhibit resistance to clinically relevant antibiotics ampicillin, penicillin, and vancomycin.

### The inhibition of L. monocytogenes by PS01155 and PS01156 in an attached-biomass assay was dependent on the microbiome context and time of incubation.

In the inhibition assay using spot inoculation, PS01155 and PS01156 successfully inhibited 22 strains of L. monocytogenes. However, the LAB strains did not significantly inhibit L. monocytogenes in the 3-day attached biomass when grown together with the environmental microbiomes collected from facility F2. Furthermore, they did not inhibit L. monocytogenes when grown under any condition for 5 or 15 days. In the 3-day assays, we observed varying reduction in L. monocytogenes concentration, depending on the microbiome context. A potential reason for this may be differences in initial levels of naturally occurring L. monocytogenes among different environmental microbiome samples. Specifically, the reduction was lowest in the presence of a microbiome from F2, which had a higher initial concentration of L. monocytogenes (3.67 *±* 0.09 log_10_ MPN/mL) than F1 (<1.52 log_10_ MPN/mL) and F3 (1.59 *±* 0.36 log_10_ MPN/mL). Further, strains PS01155 and PS01156 may have been ineffective in reducing L. monocytogenes in the 3-, 5-, and 15-day attached biomasses because of competitive exclusion under the conditions used for growing the attached biomass.

The temperature used in our experiments (15°C) is favorable for the growth of psychrotrophic bacterial families that have been previously found in tree fruit packing facilities, such as *Flavobacteriaceae*, *Pseudomonadaceae*, *Moraxellaceae*, *Xanthomonadaceae*, and *Weeksellaceae* (24). In the microbiome samples used in this study, there was a high relative abundance of Pseudomonas ASVs, which could have potentially competed for surface attachment with the lactic acid bacteria and thereby reduced their efficacy. Furthermore, bacteriocin production by lactic acid bacteria is cell population density dependent (76). The LAB strains were applied at a high concentration (~10^7^ CFU/mL), but slow growth and cell death due to competition with other microorganisms could have potentially inhibited the production of bacteriocins as well as other secondary metabolites (76, 77). Competitive exclusion may also explain why the attached biomass, grown for 3, 5, and 15 days, had significantly different microbiota compositions. Weak inhibition of L. monocytogenes may also have been observed due to our experimental design, which represented a worst-case scenario in which L. monocytogenes is present in a high concentration (~10^5^ CFU/mL), as observed in our previous study (24). Therefore, further studies are needed to determine whether PS01155 or PS01156 strains would be more effective against lower concentrations of L. monocytogenes, which may be more realistic, and whether reapplication of the strains at shorter time intervals would increase their efficacy. Lastly, all samples contained ASV1, including the negative and positive controls, suggesting that some endogenous *Enterococcus* spp. were present in the microbiome samples used in our experiments. It is possible that L. monocytogenes strains that were isolated from these same environments are coadapted to the antilisterial action of *Enterococcus*, thus decreasing the effectiveness of the added lactic acid bacteria; however, we do not have evidence to support this hypothesis.

The microbiota compositions of the experiments with different endpoints (3-day, 5-day, and 15-day experiments) were significantly different from one another, as determined by PERMANOVA, and further confirmed by differential abundance analysis. Many of the bacterial genera that were present in high relative abundance in the 3- and 5-day biofilms are composed of mesophilic bacteria that are commonly found in water, soil, air, and animals, including Pseudomonas, Acinetobacter, and *Citrobacter* (78–82). The bacteria that were present in a greater relative abundance in the 15-day biofilms contain multiple species with strong biofilm forming abilities, most notably *Pseudomonadaceae* (25–28, 30, 83) and *Flavobacteriaceae*, including species of *Myroides* (84–87). The families that were detected in a higher relative abundance in 3- and 5-day attached biomass may have been less fit to compete with the bacteria that were found at a higher relative abundance in the 15-day attached biomass. This may be due to the fact that many species of Pseudomonas are able to dominate biofilms (88–91). Both Pseudomonas and species of *Flavobacteriaceae* have been shown to enhance L. monocytogenes growth in food processing facility biofilms (30, 85, 89, 92, 93). Additionally, the presence of fungi in the environmental microbiomes of F1, F2, and F3, which were not determined in this study, could have influenced the ability of PS01155 and PS01156 to inhibit L. monocytogenes. Further research needs to be conducted to assess the effect of fungi on the antilisterial activities of the two LAB strains.

### Repeated application of PS01155 or PS01156 over 15 days did not significantly reduce L. monocytogenes concentration.

The 15-day attached-biomass assay with repeated application of PS01155 or PS01156 was performed to assess the additive effect of LAB on the L. monocytogenes concentration in attached biomass collected on day 15. However, there was no significant reduction of L. monocytogenes regardless of the source of environmental microbiota, compared to the positive control. In two studies that had applied the same PS01155 or PS01156 LAB strains in two different poultry processing facility drains, the strains were applied after cleaning and sanitizing for four contiguous days during the first week (53). Then, for the next 3 weeks, PS01155 or PS01156 was applied twice a week, and then sampling continued for up to 18 weeks after the last treatment (51, 53). In one study, modest *Listeria* sp. inhibition was observed in the first 2 weeks; however, the inhibition reached 4.1 and 2.5 log CFU/mL at the end of the study in drains at room temperatures of 30°C and 15°C, respectively (53). In the second study performed in drains at a facility processing ready-to-eat poultry, the *Listeria* spp. were undetectable in five of the six drains tested after the first week (51). In our study, attached biomass was rinsed with phosphate-buffered saline (PBS) and the two strains (PS01155 or PS01156) were reapplied on the fifth and tenth days of growth.

The results from the previous studies (51, 53) suggest that strains PS01155 and PS01156 need to be reapplied to the microbiome frequently to effectively compete with the environmental microbiota and inhibit *Listeria* spp. Furthermore, previous studies applied both strains together, indicating that the addition of both strains might have an increased ability to compete with and inhibit *Listeria* spp. (53), compared to the addition of just one of the strains to each microbiome sample in our study. In a study by Zhao et al. (51), only one of the LAB strains (strain 152, named PS01156 in this study) was found in the biofilms at the end of 8 weeks of treatment, present at a concentration of 100 CFU/cm^2^. Strains PS01155 and PS01156 had originally been applied at a concentration of ~1 × 10^7^ CFU/mL, suggesting that they did not thrive in the environment. The results from Zhao et al. (51, 53) as well as the results of our study suggest that the addition of LAB strains to food processing environments would likely need to be part of a daily sanitation routine in order to be effective. Lastly, the differences between results reported by Zhao et al. (51, 53) and our results suggest that the environmental microbiota composition as well as the concentration of L. monocytogenes may affect the antilisterial activity of lactic acid bacteria.

### Limitations.

All experiments presented here were conducted under laboratory conditions, using synthetic media that may not resemble the conditions present in tree fruit packing facilities. Additional research is therefore needed to characterize the ability of these two LAB strains to produce antilisterial compounds in conditions that more closely resemble the environments of tree fruit packing facilities. Further, rigorous safety assessments is needed to address potential health concerns of applying *Enterococcus* spp. in food processing environments.

### Conclusions.

This study has shown that the ability of LAB strains PS01155 and PS01156 to inhibit pure cultures of L. monocytogenes is not indicative of their ability to inhibit L. monocytogenes when cocultured with food processing environmental microbiomes. Future studies evaluating the efficacy of putative biocontrol strains should therefore test their efficacy not only against pure cultures of the target pathogen but also against the pathogen in the presence of an environmental microbiota that resembles the target environment in which biocontrol strains are intended to be used.

## MATERIALS AND METHODS

### Bacterial strains.

Twenty-two phylogenetically diverse Listeria monocytogenes strains previously collected from a tree fruit packing environment were included in this study (Table 2) (94). Two lactic acid bacterium strains previously identified by Zhao et al. (51) as Enterococcus durans 152 (PS01156; ATCC PTA-4759) and Lactococcus lactis subsp. *lactis* C-1-152 (PS01155; ATCC PTA-4761) were purchased from ATCC. These strains were chosen for this study because of their reported ability to inhibit L. monocytogenes (51–53). All strains were preserved at −80°C in brain heart infusion (BHI) broth (BD Life Sciences, Sparks, MD) supplemented with 20% glycerol. Before use, PS01155, PS01156, and L. monocytogenes strains were streaked from cryostock onto BHI agar (BD Life Sciences, Sparks, MD) and grown for 24 h at 35°C (for lactic acid bacteria) or 37°C (for L. monocytogenes).

### Verification of taxonomic identity of lactic acid bacteria.

Whole-genome sequencing was used to obtain a confident taxonomic identification of the LAB strains purchased from ATCC. Overnight cultures of strains PS01155 and PS01156 grown in BHI broth at 37°C were used for DNA extraction using the E.Z.N.A. bacterial DNA kit (Omega Bio-tek, Norcross, GA) following the manufacturer’s protocol. The extracted DNA was quantified using a Nanodrop One instrument (Thermo Fisher, Wilmington, DE) and Qubit 3 (Thermo Fisher, Foster City, CA) and stored at −80°C until it was sent to Novogene Bioinformatics Institute (Beijing, China) for library preparation and whole-genome sequencing. Briefly, quality and quantity of the extracted DNA were assessed using agarose gel electrophoresis to verify DNA integrity and Qubit 2.0 (Thermo Fisher, Foster City, CA) to determine DNA concentration. DNA libraries were constructed by randomly fragmenting the genomic DNA using sonication; then, fragments were end polished, A tailed, and ligated with full-length adapters for Illumina sequencing using a standard process developed by Novogene. Prepared libraries were purified with AMPure XP beads (Beckman Coulter, Indianapolis, IN), and the library fragment size distributions were verified using Agilent 2100 Bioanalyzer (Agilent Technologies, Santa Clara, CA). Libraries were quantified by real-time PCR, pooled, and sequenced on an Illumina NovaSeq 6000 (Illumina, San Diego, CA) with 150-bp paired-end sequencing. The quality of sequencing reads was assessed using FastQC v0.11.9 (95), and low-quality bases were removed with Trimmomatic v0.39 (96). Reads were assembled *de novo* using SPAdes v3.153 (97), with k-mer lengths of 99 and 127 bp (98) and the “-careful” option to reduce mismatches and short indels. The quality of the assembled reads was assessed using Quast v5.0.2 (99) by calculating assembly quality metrics, including *N*_50_, GC content, and total number of contigs. The Burrows-Wheeler Aligner tool (BWA) v0.7.17 (100) and SAMtools v1.9 (101) were used to calculate average draft genome coverage. The assembled genomes were submitted to the Type (Strain) Genome Server to identify taxonomic species (102). The confidence in taxonomic identification was evaluated based on the digital DNA-DNA hybridization (dDDH) *d*_4_ score, which indicates the sum of all identities found in high-scoring segment pairs (HSPs) divided by overall HSP length and is the relevant metric for draft assemblies (54). The BAGEL4 web server (56) was used to identify potential bacteriocin-encoding genes present in the draft genomes of lactic acid bacteria.

### Preliminary safety assessment of LAB strains.

Hemolysis, antibiotic susceptibility, and putative virulence gene detection were carried out to assess the safety of the two LAB strains. A hemolysis test was performed by streaking PS01155 and PS01156 onto tryptic soy agar (TSA) plates supplemented with 5% sheep blood (Hardy Diagnostics, Santa Maria, CA) followed by incubation at 35°C for 24 h. Bacillus cereus PS00023 was used as a positive control and Listeria innocua PS0298 was used as a negative control for hemolysis. Antibiotic susceptibility of the LAB strains was performed using Sensititre GPN3F 96-well plates (Thermo Scientific) preloaded with antibiotics. Sensititre plates were used for broth microdilution, and the MICs were interpreted using the CLSI M100-ED32 (57). A total of 18 antimicrobials were tested, including ampicillin (0.12 to 16 μg/mL), ceftriaxone (8 to 64 μg/mL), ciprofloxacin (0.5 to 2 μg/mL), clindamycin (0.12 to 2 μg/mL), daptomycin (0.25 to 8 μg/mL), erythromycin (0.25 to 4 μg/mL), gatifloxacin (1 to 8 μg/mL), gentamicin (2 to 500 μg/mL), levofloxacin (0.25 to 8 μg/mL), linezolid (0.5 to 8 μg/mL), oxacillin plus 2% NaCl (0.25 to 8 μg/mL), penicillin (0.06 to 8 μg/mL), quinupristin and dalfopristin (0.12 to 4 μg/mL), rifampin (0.5 to 4 μg/mL), streptomycin (1,000 μg/mL), tetracycline (2 to 16 μg/mL), trimethoprim-sulfamethoxazole (0.5/9.5 to 4/76 μg/mL), and vancomycin (1 to 64 μg/mL).

LAB inocula were prepared by suspending colonies of PS01155 or PS01156 (grown on BHI plates as previously described) in Mueller-Hinton (MH) broth to a final concentration of ~5 × 10^5^ CFU/mL. Fifty microliters of each culture was added per well of a Sensititre plate, including a positive control (no antibiotic added). Fifty microliters of MH broth was added to a negative-control well. Inoculated plates were covered with a sealing tape and incubated at 35°C for 18 to 24 h. To verify the inoculum concentration, dilutions of each inoculum were spread plated onto BHI agar and incubated at 35°C for 24 h. MICs were determined based on the guidelines and recommendations from CLSI, and CLSI guideline M07-A9 was utilized in instances of unclear growth interpretations (103).

To determine the presence of putative virulence factor genes in the genomes of the two LAB strains, the assembled genomes of PS01155 and PS01156 were submitted to VirulenceFinder-2.0, hosted by the Center of Genomic Epidemiology server (58).

### Spot inoculation assay.

To evaluate the antilisterial activity of PS01156 and PS01155, we performed a spot inoculation assay against 22 L. monocytogenes isolates collected from tree fruit packing facilities (Table 2) (94). L. monocytogenes isolates were previously whole-genome sequenced and reported by Chen et al. (94), and they were selected to represent a phylogenetically diverse set of strains present in tree fruit packing facilities (Table 2). LAB strains and the L. monocytogenes strains were grown on BHI agar as previously described. After incubation, colonies of L. monocytogenes isolates were suspended in 1× PBS (0.8% NaCl, 0.02% KCl, 0.144% Na_2_HPO_4_, 0.024% KH_2_PO_4_; pH adjusted to 7.4 using 1 M HCl) to an optical density at 600 nm [OD_600_] of 0.2, and diluted to concentrations of 1 × 10^8^ and 1 × 10^7^ CFU/mL. For each L. monocytogenes isolate, ~100-μL of cultures at the two concentrations were swabbed onto two separate BHI agar plates using a sterile cotton-tip swab (Puritan, Guilford, ME) to form bacterial lawns. The plates containing each L. monocytogenes lawn were spot inoculated with 1 μL of PS01156 or PS01155 inoculum, in two technical replicates, and incubated at 15, 20, 25, or 30°C for 96 h. Due to the undulate edge of the spot inoculum, zones of inhibition were measured from the outer edge of the spot to the outer edge of the zone of inhibition at three different locations. The three measurements were averaged, and the average was reported as a zone of inhibition. The experiment was performed in three independent biological replicates. One-way ANOVA was used to assess the statistical significance of L. monocytogenes inhibition by temperature and lawn concentration (α = 0.05). ANOVA and Tukey’s HSD tests were conducted with the R packages stats v4.0.3 (104) and agricolae v1.3-5 (105) in R v4.1.0 (104).

### Characterization of antilisterial activity.

To assess the effect of different growth conditions on the production of inhibitory compounds, strains PS01156 and PS01155 were grown at 37°C for 24 and 48 h in MRS broth at pH 6.2 and in BHI broth at pH 6.2 and 7 (61) (Table S2). Additionally, strain PS01156 was grown in MRS broth at 41.5°C for 12 h and at 25°C for 24 h (63), and in tryptic soy broth (TSB) at 37°C at pH 7.2 and 6.2 (62) (Table S2). After incubation, the cultures were centrifuged at 8,000 × *g* for 20 min, and the supernatant was sterilized by filtration using a 0.2-μm cellulose acetate filter (VWR, China; catalog no. 28145-477).

To evaluate the contribution of organic acids to the inhibition of L. monocytogenes, the filtered supernatant was neutralized to pH 7 using 1 M NaOH. To evaluate the relative contribution of hydrogen peroxide to the inhibition of L. monocytogenes, the neutralized supernatant was treated with 1 mg/mL catalase from bovine liver (Sigma-Aldrich, St. Louis, MO) for 30 min at 25°C. To determine whether the antilisterial compounds are proteinaceous, the catalase-treated supernatant was treated with 1 mg/mL proteinase K (VWR Chemicals, Radnor, PA) for 2 h at 37°C.

A disk diffusion assay was performed to assess the ability of treated supernatants to inhibit L. monocytogenes isolate PS01273 lawns (~1 × 10^7^ CFU/mL), prepared as previously described. An aliquot of 25 μL of each treated supernatant (i.e., filtered, pH neutralized, catalase treated, and proteinase K treated) was applied to a sterile disk (Hardy Diagnostics, Santa Maria, CA) and placed over a lawn of L. monocytogenes. Lawns were incubated at 30°C for 24 or 48 ± 2 h. Inhibition zones were measured from the outer edge of the disk to the outer edge of the zone of inhibition. One-way ANOVA was used to assess the statistical significance of L. monocytogenes inhibition by supernatant treatment (α = 0.05). ANOVA and Tukey’s HSD tests were conducted with the R packages stats v4.0.3 (104) and agricolae v1.3-5 (105) in R v4.1.0 (104). The incubation temperature, pH, and medium in which strains PS01156 and PS01155 produced the largest quantities of inhibitory compounds after a 24-h incubation were used to grow the PS01156 and PS01155 strains for the attached-biomass assay described below.

### Environmental microbiome collection.

Environmental microbiome samples were collected on two visits (4 April 2019 and 24 April 2019) to three packing facilities located in the northeastern United States. Samples were collected underneath the roller brush conveyor in the washing, drying, and waxing sections of the packing lines. Samples were collected using three hydrated sponges with a neutralizing broth (3M, St. Paul, MN) from a 40- by 40-cm surface and transported to the lab on ice. Ninety milliliters of BHI broth was added to each sampling bag followed by stomaching for 7 min at 260 rpm to release the cells from the sampling sponge. All samples collected from the same facility were combined to create a composite sample representative of each facility. Composite samples were supplemented with 20% (vol/vol) sterile glycerol, thoroughly mixed, aliquoted in five 50-mL conical tubes (VWR, Radnor, PA), and stored at −80°C until further use. The frozen microbiome samples were thawed at room temperature for 1.5 h prior to use in subsequent experiments.

### Attached-biomass assay.

The attached-biomass assay was developed as an *in vitro* model system to determine the effects of the presence of the environmental microbiome of tree fruit packing facilities on the antilisterial properties of PS01155 and PS01156. The assay was developed, and standardized by following laboratory practices used to grow biofilms *in vitro* (106). However, since we quantified only microorganisms that attached to the surface, without assessing the formation of extracellular polymeric substances that characterize biofilms, we refer to this assay as “attached-biomass assay.” Attached biomass was grown in 15-mL polypropylene conical tubes (VWR, Radnor, PA) which provided single-use, clean, unscathed surfaces to limit the impact of external, uncontrolled factors on the formation of attached biomass (107). One isolated colony from PS01155 or PS01156 grown on a BHI plate as previously described was suspended in MRS broth (pH 6.2) and grown for 24 h at 37°C (61). After incubation, the concentration of lactic acid bacteria was adjusted to ~1 × 10^8^ CFU/mL. One isolated colony from L. monocytogenes isolate PS01273 grown on a BHI plate as previously described was streaked onto BHI agar and incubated for 24 h. Colonies of L. monocytogenes were suspended in PBS to an OD_600_ of ~0.2 and diluted 100-fold to a final concentration of ~1 × 10^6^ CFU/mL. Each experiment included a negative control (environmental microbiome composite sample in BHI), a positive control (environmental microbiome composite sample in BHI and ~1 × 10^5^ CFU/mL L. monocytogenes), a PS01155 treatment or a PS01156 treatment (environmental microbiome sample in BHI, ~1 × 10^5^ CFU/mL L. monocytogenes, and ~1 × 10^7^ CFU/mL of PS01155 or PS01156), and a sterility control (sterile BHI). All sample tubes contained a final volume of 2 mL.

To evaluate whether the time of incubation of attached biomass influences the inhibition of L. monocytogenes by the LAB strains, attached-biomass experiments were incubated at 15°C for 3 days, 5 days, and 15 days. In the 3- and 5-day experiments, PS01155 or PS01156 was added on day 0; while in the 15-day experiment, PS01155 or PS01156 was added on day 0 and reapplied on days 5 and 10 to evaluate whether reapplication resulted in additional reduction of L. monocytogenes. On days 5 and 10 of the 15-day experiment, detached cells were removed, and the attached biomass was washed twice with sterile PBS. PS01155 or PS01156 was prepared and reapplied at the same concentration as on day 0. No reapplication was done for the positive- and negative-control samples. Each assay was conducted in three independent biological replicates.

### Quantification of aerobic mesophilic microorganisms and L. monocytogenes.

On day 0, environmental microbiome composite samples were serially 10-fold diluted in PBS for aerobic plate count (APC) and L. monocytogenes enumeration using the MPN assay described below. On the last day of incubation (i.e., day 3, 5, or 15), the detached cells were removed from the tubes and the attached biomass was washed twice with 2 mL of sterile PBS (106). The attached biomass was then detached by adding 2 mL of PBS and 1 g of 3-mm sterile glass beads (MP Biomedicals, Hessen, Germany), followed by vortexing for 2.5 min. One milliliter of released attached biomass was used for DNA extraction, and the remaining volume was serially 10-fold diluted in PBS for total aerobic plate count and L. monocytogenes quantification.

For total aerobic plate count, sample dilutions were spread plated in triplicate onto BHI agar plates and incubated at 37°C for 48 ± 2 h. BHI agar and an incubation temperature of 37°C were chosen over the conditions of the standard plate count method (108) due to better growth of the aerobic mesophilic microorganisms present in the environmental samples, as determined in preliminary experiments (data not shown).

L. monocytogenes was enriched and quantified according to the Food and Drug administration (FDA) Bacteriological Analytical Manual (BAM) methods for detection and enumeration of L. monocytogenes (109) and for determining MPN from serial dilutions (110). Briefly, 100 μL of each dilution was inoculated into three sterile microcentrifuge tubes prefilled with 900 μL of buffered *Listeria* enrichment broth (BLEB) (Hardy Diagnostics, Santa Maria, CA). Inoculated microcentrifuge tubes were incubated at 30°C. After 4 h of incubation, 4 μL of *Listeria* selective supplement (10 mg/L acriflavine, 50 mg/L nalidixic acid, and 40 mg/L cycloheximide; Sigma-Aldrich, St. Louis, MO) was added to each tube, followed by incubation at 30°C for an additional 44 ± 2 h. After incubation, one loopful of each MPN tube was streaked onto agar *Listeria* Ottaviani & Agosti (ALOA) agar plates (Bio-Rad, Marnes la Coquette, France). Inoculated plates were incubated for 24 to 48 h at 37°C. After incubation, ALOA plates were examined for growth of blue-green colonies with a halo, which are characteristic of L. monocytogenes. The BAM MPN calculator (110) was used to calculate the MPN/sample. The significance of aerobic plate counts and L. monocytogenes quantification for each assay was assessed for each incubation time after log_10_ transformation by performing a one-way ANOVA and Tukey’s HSD test using the R packages stats v4.0.3 (104) and agricolae v1.3-5 (105) in R v4.1.0 (104).

### DNA extraction for microbiota sequencing.

One milliliter of each released attached biomass sample was centrifuged at 13,000 × *g* for 20 min (Eppendorf, Hamburg, Germany) to pellet all the cells. The supernatant was discarded, and the pellets were stored at −80°C until DNA extraction using DNeasy Power biofilm kit (Qiagen, Germantown, MD) following the manufacturer’s protocol. The extracted DNA was quantified spectrophotometrically using a Nanodrop One instrument (Thermo Fisher, Wilmington, DE) and fluorometrically using Qubit 3 (Thermo Fisher, Foster City, CA), with a double-stranded-DNA (dsDNA) high-sensitivity assay kit (Thermo Fisher, Foster City, CA). Extracted DNA samples were stored at −80°C until further use.

### 16S rRNA gene V4 amplification, amplicon library preparation, and amplicon sequencing.

Amplification of the 16S rRNA gene V4 region, library preparation, and sequencing were carried out by Novogene Bioinformatics Institute (Beijing, China). Briefly, 16S rRNA gene V4 PCR amplification was performed by using forward primer 515F and reverse primer 806R (111–113) with Illumina barcodes. DNA libraries were constructed by end repairing and adding As to tails, followed by purification. The DNA libraries were pooled and sequenced using an Illumina NovaSeq 6000 system (Illumina, San Diego, CA) to generate 250-bp paired-end reads.

### Bioinformatic analyses.

Sequences were analyzed with the DADA2 v3.14 pipeline following the standard protocol for 16S rRNA V4 region amplicon sequence reads in R (114). Low-quality sequence reads were removed, and low-quality bases were trimmed from reads. Error rates were calculated for the data set and the ASVs were inferred. Paired-end sequence variants were merged, and sequences shorter than 251 or longer than 253 bp were discarded. Chimeras were detected and removed from the data set, and remaining ASVs were assigned taxonomy using the reference database Silva (v132) (115).

### ASV normalization and PCA.

The ASV table was normalized using the compositional analysis approach (116). First, zeros were replaced with a small nonzero value using the R package zCompositions v1.3.4 (117). The ASV table was normalized using center log-ratio (CLR) transformation. Singular value decomposition was then used to perform PCA to evaluate whether samples cluster by facility, growth time (3-day, 5-day, or 15-day experiment), or treatment (with and without the addition of strain PS01155 or PS01156). Additionally, relative abundance was calculated from CLR transformation-normalized abundances using the Aitchison simplex method using the R package Compositions v2.0-1 (118). Stacked bar plots were used to visualize the most abundant taxa in each growth time experiment and facility sample using the R package ggplot2 v3.3.5 (119).

### Statistical analysis and differential abundance analysis.

PERMANOVA was performed based on Aitchison distances to test whether there were significant differences in the microbiota composition between samples collected at experiment end points from (i) different attached-biomass growth time experiments, (ii) different facilities, and (iii) different treatments (negative control, positive control, PS01155, or PS01156). PERMANOVA was carried out using the R package pairwiseAdonis v0.0.1 (120). Differential abundance (DA) analysis was used to identify ASVs that were differentially abundant in samples grown in 3- versus 5-day attached biomass, 5- day versus 15-day attached biomass, and 3-day versus 15-day attached biomass. The R package ALDEx2 v1.24.0 (121) was used to calculate differential abundance using default parameters at the ASV level. This method was chosen because it is appropriate for compositional data analysis and has been shown to minimize the false discovery rate (FDR) (121). Significantly differentially abundant taxa were identified based on the Welch’s *t* test and Wilcoxon rank-sum test, with a Benjamini-Hochberg correction of a *P* value of <0.05, followed by the application of an effect size cutoff of |1| as suggested by the program manual (121).

### Data availability.

Whole-genome sequence reads of PS01155 and PS01156 were deposited in NCBI under BioProject no. PRJNA670330 with sequence accession no. SAMN16493025 and SAMN16493026, respectively. Assembled genomes were deposited in GenBank under accession no. SAMN28178373 (PS01155) and SAMN28178374 (PS01156). Sequencing reads of the attached microbiotas from attached biomass experiments were deposited in NCBI under accession no. PRJNA813407. All code used for the analyses reported in this study is available at https://github.com/LauRolon/SHAP.
