# Supplementary material for: Ability of Two Strains of Lactic Acid Bacteria To Inhibit Listeria monocytogenes by Spot Inoculation and in an Environmental Microbiome Context
Source: Microbiol Spectr. 2022 Jul 19;10(4):e01018-22. doi: 10.1128/spectrum.01018-22 (PMC9431016; doi:10.1128/spectrum.01018-22)
Supplement: Supplemental file 1 — Supplemental material. Download spectrum.01018-22-s0001.pdf, PDF file, 0.2 MB [file spectrum.01018-22-s0001.pdf]

Supplemental Material

The ability of two lactic acid bacteria strains to inhibit *L. monocytogenes* by spot-inoculation and in an environmental microbiome context

Priscilla Sinclair[a*], M. Laura Rolon[a,b*], Jingzhang Feng[a], Adrián F. Padín-López[a], Luke LaBorde[a], Jasna Kovac[a,b]

[a]Department of Food Science, The Pennsylvania State University, University Park, PA 16802

[b]Microbiome Center, The Pennsylvania State University, University Park, PA 16802

[*]Equal contribution

TABLE S1 Locus tags of bacteriocin encoding genes detected in the draft genomes of strains PS01155 and PS01156 using BAGEL 4.

**Strain**	**Bacteriocin gene**	**Locus tag**	**Start**	**End**	**Accession**	**E-value**	**Query coverage (%)**	**Identity (%)**
PS01155	Enterocin B	NODE_29_length_12683_cov_51.018079	1,978	12,682	WP_002295295.1	9×10^{-33}	100	100
	Enterocin P	NODE_18_length_47365_cov_62.837059	36,713	47,365	WP_010733280.1	4×10^{-39}	100	98.51
	Enterolysin A	NODE_18_length_47365_cov_62.837059	17,171	37,714	WP_005877003.1	0.0	100	100
	Enterocin A	NODE_10_length_102565_cov_59.833919	91,889	102,565	WP_002304799.1	1×10^{-38}	100,	100
PS01156	Enterocin P	NODE_39_length_10873_cov_57.676717	0	10,872	WP_002291094.1	6×10^{-43}	100	100
	Enterocin L50b	NODE_53_length_6845_cov_39.432569	0	6,844	WP_002293183.1	8×10^{-22}	100	100
	Enterocin L50a	NODE_53_length_6845_cov_39.432569	0	6,844	WP_236918740.1	3×10^{-28}	50	100
	Enterolysin A	NODE_21_length_43187_cov_64.562494	17,087	37,630	WP_002293508.1	0	100	100
	UviB	NODE_8_length_118611_cov_59.795483	62,363	82,507	P_002342148.1	1×10^{-41}	100	100

TABLE S2 Growth conditions used for characterization of antilisterial activity of lactic acid bacteria strains PS01155 and PS01156.

Strain	Medium[a]	pH	Temperature (°C)	Time (h)
PS1155	MRS	6.2	37	24
				48
	BHI	6.2	37	24
				48
	BHI	7.0	37	24
				48
PS1156	MRS	6.2	37	24
				48
			41.5	12
			25	24
	BHI	6.2	37	24
			37	48
	BHI	7.0	37	24
			37	48
	TSB	6.2	37	24
		7.0	37	24

[a]MRS, deMann, Rogosa, and Sharp broth; BHI, Brain Heart Infusion broth; TSB, Tryptic Soy Broth.
